# IMPLEMENTING THE MEDICARE ANNUAL WELLNESS VISIT: A GWEP COLLABORATION

**DOI:** 10.1093/geroni/igz038.1445

**Published:** 2019-11-08

**Authors:** Ellen Flaherty, Nina Tumosa

**Affiliations:** 1 Dartmouth-Hitchcock Medical Center, Lebanon, New Hampshire, United States; 2 Health Resources and Services Administration, Rockville, Maryland, United States

## Abstract

The Geriatric Interprofessional Team Transformation in Primary Care (GITT-PC) model improves delivery of healthcare to older adults in primary care by training healthcare professionals in team functioning, rapid cycle QI, and evidence based geriatric practice. The program capitalizes on the role of nursing and other healthcare disciplines. To maximize sustainability, it focuses on Medicare-reimbursable visits. This program will focus on the implementation of the Annual Wellness Visit (AWV) through a collaboration between Dartmouth and 3 GWEPs: University of Florida, University of Louisville and the University of Wyoming. The model’s standardized approach to implementation begins with practice assessments and two trainings. The first training focuses on team functioning & rapid cycle QI and the second is a deep dive training focused on the implementation of the AWV. After the training, practices participate in a data-driven, virtual learning collaborative with monthly data collection and learning sessions. Since 2015, the AWV, through the GITT-PC model has been implemented in 14 sites in northern New England, 10 sites in upstate New York, and nationally through five other GWEP awardees across the country.

